# Supplementary material for: Fleas of black rats (Rattus rattus) as reservoir host of Bartonella spp. in Chile
Source: PeerJ. 2019 Aug 1;7:e7371. doi: 10.7717/peerj.7371 (PMC6679904; doi:10.7717/peerj.7371)
Supplement: Supplemental Information 2 [file peerj-07-7371-s002.pdf]

Supplement file 1. AlignmentGLTA.fasta

```
>II124a_Bart_gltA
ATTTGCATGTATCGCAGCAGGTGTTGCATGTCTTTGGGGACCAGCGCATG
GAGGAGCTAATGAAGCATGCTTAAAAATGCTCCAAG---AAAT-AGGTTC
TGTGAAAAAAATTCCTGAATTTATTACTC-----GTGCAAA
>III57a_Bart_gltA
GTTTGCATGCATTGCTGCAGGTGTTGCTTGTCTTTGGGGACCAGCGCATG
GAGGAGCTAATGAAGCATGTCTGAAAATGCTACAAG---AAAT-AGGGGA
CGTTAAAAGAATTCCTGAATTTATTGCGC-----GTGCCAA
>III81d_Bart_gltA
GTTTGCGTGTATTGCAGCAGGTGTTGCCTGTCTTTGGGGACCTGCACATG
GGGGTGCCAATGAAGCCTGCTTAAAAATGTTACAAG---AAAT-AGGCTC
AGTTGAGCAAATTCCAAAATTCATTGCAC-----GAGCAAA
>III82d_Bart_gltA
GTTTGCGTGTATTGCAGCAGGTGTTGCCTGTCTTTGGGGACCTGCACATG
GGGGTGCCAATGAAGCCTGCTTAAAAATGTTACAAG---AAAT-AGGCTC
AGTTGAGCAAATTCCAAAATTCATTGCAC-----GAGCAAA
>IV160a_Bart_gltA
GTTTGCCTGTATTGCAGCAGGTGTTGCATGCCTTTGGGGACCAGCACATG
GTGGCGCCAATGAAGCATGTCTAAAAATGCTACAGG---AAAT-TGGTTC
TATTAAAAGAATTCCTGAATTTATTGCTC-----GTGCAAA
>IV160e_Bart_gltA
GTTTGCCTGTATTGCAGCAGGTGTTGCATGCCTTTGGGGACCAGCCCATG
GTGGTGCCAATGAAGCATGCCTAAAAATGCTACAAG---AGAT-AGGTTC
TATTAAAAGAATTCCTGAATTTATTGCTC-----GTGCAAA
>IV160j_Bart_gltA
GTTTGCCTGTATTGCAGCAGGTGTTGCATGCCTTTGGGGACCAGCACATG
GTGGCGCCAATGAAGCATGTCTAAAAATGCTACAGG---AAAT-TGGTTC
TATTAAAAGAATTCCTGAATTTATTGCTC-----GTGCAAA
>IV160k_Bart_gltA
GTTTGCCTGTATTGCAGCAGGTGTTGCATGCCTTTGGGGACCAGCACATG
GTGGCGCCAATGAAGCATGTCTAAAAATGCTACAGG---AAATATGGTTC
TATTAAAAGAATTCCTGAATTTATTGCTC-----GTGCAAA
>IV70a_Bart_gltA
GTTTGCGTGTATTGCAGCAGGTGTTGCCTGTCTTTGGGGACCTGCACATG
GGGGTGCCAATGAAGCCTGCTTAAAAATGTTACAAG---AAAT-AGGCTC
AGTTGAGCAAATTCCAAAATTCATTGCAC-----GAGCAAA
>IV70b_Bart_gltA
GTTTGCGTGTATTGCAGCAGGTGTTGCCTGTCTTTGGGGACCTGCACATG
GGGGTGCCAATGAAGCCTGCTTAAAAATGTTACAAG---AAAT-AGGCTC
AGTTGAGCAAATTCCAAAATTCATTGCAC-----GAGCAAA
>IV70e_Bart_gltA
GTTTGCGTGTATTGCAGCAGGTGTTGCCTGTCTTTGGGGACCTGCACATG
GGGGTGCCAATGAAGCCTGCTTAAAAATGTTACAAG---AAAT-AGGCTC
AGTTGAGCAAATTCCAAAATTCATTGCAC-----GAGCAAA
>IV77a_Bart_gltA
GTTTGCGTGTATTGCAGCAGGTGTTGCCTGTCTTTGGGGACCTGCACATG
GGGGTGCCAATGAAGCCTGCTTAAAAATGTTACAAG---AAAT-AGGCTC
AGTTGAGCAAATTCCAAAATTCATTGCAC-----GAGCAAA
>IV81b_Bart_gltA
GTTTGCGTGTATTGCAGCAGGTGTTGCCTGTCTTTGGGGACCTGCACATG
GGGGTGCCAATGAAGCCTGCTTAAAAATGTTACAAG---AAAT-AGGCTC
AGTTGAGCAAATTCCAAAATTCATTGCAC-----GAGCAAA
```

```
>V23a_Bart_gltA
GTTTGCTTGTATTGCAGCAGGTGTTGCATGCCTTTGGGGACCCGCACATG
GTGGTGCCAATGAAGCATGTCTAAAAATGCTACAAG---AAAT-AGGTTC
TATTAAAAGAATTCCTGAATTTATTGCAC-----GTGCAAA
>V23c_Bart_gltA
GTTTGCTTGTATTGCAGCAGGTGTTGCATGCCTTTGGGGACCCGCACATG
GTGGTGCCAATGAAGCATGTCTAAAAATGCTACAAG---AAAT-AGGTTC
TATTAAAAGAATTCCTGAATTTATTGCAC-----GTGCAAA
```
