# Supplementary material for: Fleas of black rats (Rattus rattus) as reservoir host of Bartonella spp. in Chile
Source: PeerJ. 2019 Aug 1;7:e7371. doi: 10.7717/peerj.7371 (PMC6679904; doi:10.7717/peerj.7371)
Supplement: Supplemental Information 3 [file peerj-07-7371-s003.pdf]

Supplement file 2. AlignmentRPOB.fasta

```
>II124a_Bart_rpoB
AAACAGCTTGTTTCAGTAGCTGCTGCGCTTATTCCATTTTTAGAAAATGA
TGATGCGAATCGTGCATTGATGGGATCAAATATGCAGCGTCAGGC
>III57a_Bart_rpoB
AAGCAGTTGGTTTCAGTAGCAGCAGCTCTTATTCCATTTTTGGAAAATGA
TGATGCGAATCGTGCATTGATGGGATCAAACATGCAACGTCAGGC
>III81d_Bart_rpoB
AAGCAGTTGGTTTCTGTGGCAGCTGCTCTTATTCCGTTTTTGGAAAATGA
TGATGCGAATCGTGCGTTGATGGGATCAAATATGCAGCGTCAAGC
>III82d_Bart_rpoB
AAGCAGTTGGTTTCTGTGGCAGCTGCTCTTATTCCGTTTTTGGAAAATGA
TGATGCGAATCGTGCGTTGATGGGATCAAATATGCAGCGTCAAGC
>IV160a_Bart_rpoB
AAACAGTTGGTTTCAGTTGCAGCTGCGCTTATTCCCTTTTTGGAAAATGA
TGATGCGAATCGTGCGTTGATGGGATCAAACATGCAGCGTCAGGC
>IV160e_Bart_rpoB
AAACAGTTGGTTTCGGTTGCAGCCGCACTTATTCCGTTTTTAGAAAATGA
TGATGCGAATCGTGCGCTTATGGGATCGAACATGCAGCGTCAGGC
>IV160j_Bart_rpoB
AAACAGTTGGTTTCAGTTGCAGCTGCGCTTATTCCCTTTTTGGAAAATGA
TGATGCGAATCGTGCGTTGATGGGATCAAACATGCAGCGTCAGGC
>IV160k_Bart_rpoB
AAACAGTTGGTTTCAGTTGCAGCTGCGCTTATTCCCTTTTTGGAAAATGA
TGATGCGAATCGTGCGTTGATGGGATCAAACATGCAGCGTCAGGC
>IV70a_Bart_rpoB
AAGACGTTGGTTTCTGTGGCAGCTGCTCTTATTCCGTTTTTGGAAAATGA
TGATGCGAATCGTGCGTTGATGGGATCAAATATGCAGCGTCAAGC
>IV70b_Bart_rpoB
AAGCAGTTGGTTTCTGTGGCAGCTGCTCTTATTCCGTTTTTGGAAAATGA
TGATGCGAATCGTGCGTTGATGGGATCAAATATGCAGCGTCAAGC
>IV70e_Bart_rpoB
AAGCAGTTGGTTTCTGTGGCAGCTGCTCTTATTCCGTTTTTGGAAAATGA
TGATGCGAATCGTGCGTTGATGGGATCAAATATGCAGCGTCAAGC
>IV77a_Bart_rpoB
AAGCAGTTGGTTTCTGTGGCAGCTGCTCTTATTCCGTTTTTGGAAAATGA
TGATGCGAATCGTGCGTTGATGGGATCAAATATGCAGCGTCAAGC
>IV81b_Bart_rpoB
AAGCAGTTGGTTTCTGTGGCAGCTGCTCTTATTCCGTTTTTGGAAAATGA
TGATGCGAATCGTGCGTTGATGGGATCAAATATGCAGCGTCAAGC
>V23a_Bart_rpoB
AAACAGTTGGTTTCAGTTGCAGCTGCGCTTATTCCCTTTTTGGAAAATGA
TGATGCGAATCGTGCACTGATGGGATCAAACATGCAGCGTCAGGC
>V23c_Bart_rpoB
AAACAGTTGGTTTCAGTTGCAGCTGCGCTTATTCCCTTTTTGGAAAATGA
TGATGCGAATCGTGCACTGATGGGATCAAACATGCAGCGTCAGGC
```
